# Differences in Physical and Psychosocial Characteristics Between CFS and Fatigued Non-CFS Patients, a Case-Control Study

**DOI:** 10.1007/s12529-016-9544-0

**Published:** 2016-02-19

**Authors:** Veronique De Gucht, Franshelis Katerinee Garcia, Marielle den Engelsman, Stan Maes

**Affiliations:** Health, Medical, and Neuropsychology Unit, Leiden University, Wassenaarseweg 52, P.O. BOX 955, 2300 RB Leiden, Netherlands

**Keywords:** Chronic fatigue syndrome, Fatigue, Matched case-control, Illness representations, Behavioral regulation pattern, Psychosocial characteristics

## Abstract

**Purpose:**

The main research question is: “Do CFS patients differ from fatigued non-CFS patients with respect to physical, cognitive, behavioral, social, and emotional determinants?” In addition, group differences in relevant outcomes were explored.

**Method:**

Patients who met the Centers for Disease Control (CDC) criteria for CFS were categorized as CFS; these patients were mainly recruited via a large Dutch patient organization. Primary care patients who were fatigued for at least 1 month and up to 2 years but did not meet the CDC criteria were classified as fatigued non-CFS patients. Both groups were matched by age and gender (*N* = 192 for each group).

**Results:**

CFS patients attributed their fatigue more frequently to external causes, reported a worse physical functioning, more medical visits, and a lower employment rate. The results of a multiple logistic regression analysis showed that patients who believe that their fatigue is associated with more severe consequences, that their fatigue will last longer and is responsible for more additional symptoms are more likely to be classified as CFS, while patients who are more physically active and have higher levels of “all or nothing behavior” are less likely to be classified as having CFS.

**Conclusion:**

A longitudinal study should explore the predictive value of the above factors for the transition from medically unexplained fatigue to CFS in order to develop targeted interventions for primary care patients with short-term fatigue complaints.

## Introduction

Fatigue can be defined as “a sense of physical tiredness and lack of energy, different from sadness and weakness” [1]. About 18 to 39 % of the general adult population experiences fatigue [2–5]. Fatigue can be seen as a continuum, ranging from mild complaints to severe disabling and persisting fatigue, like chronic fatigue syndrome (CFS) [6].

CFS is a chronic condition that is, according to the Centers for Disease Control (CDC) criteria, characterized by unexplained, severe, and persistent fatigue lasting for at least 6 months; the fatigue is not due to ongoing exertion or to a medical condition, is not improved by rest and may be worsened by physical or mental exertion, significantly interferes with daily activities and work, and is accompanied by four or more out of eight other somatic symptoms [7]. The prognosis of CFS is often poor with less than 10 % returning to premorbid levels of functioning if the condition is left untreated [8].

Several studies have shown that CFS patients differ from fatigued non-CFS patients [9–11]. Fatigue in CFS patients is often more severe than in fatigued non-CFS patients, and CFS patients report more somatic symptoms. CFS patients more frequently believe that their fatigue will last for a very long time and will be associated with more adverse consequences, but CFS patients and fatigued non-CFS patients do not seem to differ in perceptions of control over their fatigue [9]. Furthermore, studies have shown that CFS patients attribute their illness rather to external physical causes than to internal or psychological causes [12, 13], and their levels of psychological distress are higher [9–11]. In terms of outcomes, CFS patients are found to be less able to conduct everyday activities, their level of physical functioning is lower, they visit more frequently their general practitioner (GP), have a higher absenteeism from work, and a lower employment rate [9–11]. Each of the above studies has, however, included only a limited set of determinants and varied importantly in terms of the comparison group that was used, depending upon the research question.

As it proves to be very difficult to influence CFS once it is firmly established, efforts should be directed at interventions at an earlier stage, targeting determinants that discriminate significantly between a non-CFS group and a CFS group, as these factors could also be responsible for a transition from short-term fatigue to CFS. The present study aims at identifying such determinants. The broad range of physical, cognitive, behavioral, social, and emotional determinants included in the present study was selected based on reviews of factors that may play a role in the development and maintenance of CFS [14–17] and the comparative studies cited above [9–13].

The present study is a matched case-control study including a broad set of modifiable determinants and some relevant outcomes. The central research question is: Do CFS patients, who meet the CDC criteria, differ from fatigued non-CFS patients, reporting fatigue for at least 1 month up to 2 years, with respect to physical (sleep, physical activity), cognitive (illness representations), behavioral (all or nothing and limiting behavior), social (social support), and emotional determinants (anxiety, depression)? In addition, group differences in a number of relevant outcomes (physical functioning, work status, and medical visits) were explored.

## Methods

### Participants and Procedure

CFS patients were recruited from a large Dutch patient organization and filled in an online questionnaire. Patients reporting fatigue of at least 1-month (but less than 2 years) duration were recruited via primary health care centers and completed the same online questionnaire. All participants signed an informed consent before taking part in the study. Participants who had a concurrent somatic condition that could explain the fatigue symptoms or those who had a severe psychiatric disorder were excluded from the study. The inclusion or exclusion was done on the basis of self-report.

A total of 723 patients completed the questionnaire. One-hundred and twenty-one participants were excluded due to missing data and 39 due to the presence of a somatic condition and/or psychiatric disorder. The final sample consisted of 563 participants. Participants were then categorized as CFS cases if they met the CDC criteria for CFS. Participants who reported fatigue for at least 1 month but did not meet the CDC criteria for CFS were categorized as fatigued non-CFS cases. The CDC criteria were assessed on the basis of a self-report questionnaire [18]. A total of 267 were categorized as CFS cases (202 of the patient organization and 65 of the primary care group) and 296 as fatigued non-CFS cases (all from the primary care group). Both groups were subsequently matched by age and gender using the SPSS plugin FUZZY. The final matched sample consisted of 192 CFS and 192 fatigued non-CFS cases. The mean age of the CFS group was 40.50 (SD = 12.20), and the mean age of the fatigued non-CFS group was 40.22 (SD = 12.20). Both groups consisted of 22 males (11.5 %) and 170 females (88.5 %).

## Measures

### Patient Characteristics Include *Age*, *Gender*, *and Educational Level*

#### Fatigue and Somatic Complaints

*Fatigue severity* was assessed using the *subjective experience of fatigue* subscale of the Checklist Individual Strength (CIS-20R) [19] with higher scores indicating more fatigue.

*Fatigue duration* was assessed by means of self-report (number of weeks/months fatigued).

*Somatic complaints* were assessed with the Patient Health Questionnaire-15 [20]. Higher scores indicate more somatic complaints.

#### Determinants

*Sleep* was measured by asking participants to indicate the average amount of hours they sleep per night.

*Physical activity* levels were measured using the Short Questionnaire to Asses Health Enhancing Physical Activity [21]. Higher scores indicate more physical activity.

*Patients’ illness representations* were assessed using the Brief Illness Perception Questionnaire (Brief IPQ) [22]. Higher scores on the dimensions consequences, timeline, identity, and emotional representation indicate more severe consequences, a longer expected fatigue duration, more additional symptoms, and more emotional consequences. Higher scores on personal control, treatment control, and understanding indicate higher beliefs in personal or treatment control and more understanding of one’s fatigue. For the cause dimension, patients were given a list of nine potential causes and had to indicate for each of these causes if they considered it to be a potential cause of their fatigue/CFS or not (making use of a yes/no scale).

*Behavioral regulation patterns* were measured using the “all or nothing” (a pattern of over-activity followed by rest) and “limiting behavior” (reducing daily activities and excessive resting) scales of the Behavioral Reponses to Illness Questionnaire (BRIQ) [23]. Higher scores indicate more “all or nothing” or “limiting behavior.”

*Social support* was measured with two questions: “How much social support do you receive from your partner (best friend or family member) for your fatigue?” and “How much social support do you receive from others who are not your partner (best friend or family member)?,” rated on a 5-point Likert scale ranging from ‘no support’ to “a lot of support.”

*Psychological distress* was assessed with the anxiety and depression subscales of the Brief Symptom Inventory (BSI) [24].

#### Outcome Variables

*Physical functioning* was assessed using the Short Form Health Survey (SF-12 v2) [25]. Lower scores indicate worse physical functioning.

*Employment status and medical visits* during the past 6 months were assessed by self-report.

### Statistical Analysis

First, comparisons between the CFS and the fatigued non-CFS group were made using independent *t* tests for normally distributed continuous variables, Mann–Whitney U tests for non-normally distributed continuous variables, and *χ*2 for categorical variables. To reduce the chances of obtaining false-positive results, due to multiple comparisons, a Bonferroni correction was used. The Bonferroni-corrected alpha level that corresponds with an alpha level of 0.05 was 0.002 (0.05/30). All *p* values lower or equal to 0.002 were considered to be significant. All tests performed were two-tailed tests.

Next, a multivariate logistic regression analysis was conducted to identify which determinants contributed most to the likelihood of being diagnosed with CFS. The analyses were conducted using SPSS v21 for windows.

## Results

Table 1 shows the results of the comparative tests. With regard to patient characteristics, no significant differences were found for age or gender, as subjects were matched for both age and gender. No significant difference was found for educational level. CFS patients reported a significantly longer duration of fatigue, a higher fatigue severity, and more somatic complaints than fatigued non-CFS patients.

**Table 1:** Descriptives of and differences between the CFS and fatigued non-CFS patients

Variable	Non-CFS *N* = 192	CFS *N* = 192	Test for differences	95 % CI^**a**^	*p*	Effect size *r*
Patient characteristics						
Age, years	40.22 ± 12.08	40.50 ± 12.20	*t* = −.227	−2.72, 2.16	.821	–
Gender (female)	170 (88.5)	170 (88.5)	–	–		
Educational level (N, %)			*χ* ^*2*^ = 4.63	–	.111	–
Primary education^b^	1 (0.5)	7 (3.6)				
Secondary education	53 (27.6)	53 (27.6)				
Higher education	138 (71.9)	132 (68.8)				
Fatigue and somatic complaints						
Fatigue severity	43.97 ± 6.81	47.83 ± 7.31	*U* = 12176.5	–	*.000*	−.29
Fatigue duration, weeks	40.30 ± 30.93	454.85 ± 459.52	*U* = 4393	–	*.000*	−.66
Somatic complaints	11.07 ± 4.50	14.23 ± 4.54	*t* = −6.86	−4.07, 2.26	*.000*	−.33
Determinants						
Average hours of sleep per night ^c^	7.01 ± 1.43	7.55 ± 1.81	*t* = −3.25	−0.87, −0.21	*.001*	−.16
Minutes of PA per week	221.68 ± 304.67	154.98 ± 541.20	*U* = 12064.5	–	*.000*	.30
Illness representations						
Consequence	6.93 ± 1.73	8.50 ± 1.57	*U* = 8981.5	–	*.000*	−.41
Timeline	5.39 ± 1.99	7.57 ± 1.95	*t* = −10.86	−2.58, −1.79	*.000*	−.48
Personal control	4.48 ± 2.00	4.13 ± 2.13	*t* = 1.68	−0.06, 0.77	.094	–
Treatment control	3.44 ± 2.56	4.47 ± 2.58	*t* = −3.95	−1.55, −0.52	*.000*	−.20
Identity	5.33 ± 2.24	7.41 ± 2.08	*U* = 8510	–	*.000*	−.05
Understanding	6.23 ± 2.43	5.71 ± 2.65	*t* = 1.99	0.01, 1.03	.048	–
Emotional representation^c^	6.01 ± 1.65	6.34 ± 2.02	*t* = −1.74	−1.40, −0.08	.083	–
Attributions of fatigue (*N*, %)						
A germ or virus	35 (18.2)	120 (62.5)	*χ* ^*2*^ = 78.16	–	*.000*	−.45
Diet or eating habits	75 (39.1)	63 (32.8)	*χ* ^*2*^ = 1.63	–	.202	–
Pollution	31 (16.1)	31 (16.1)	*χ* ^*2*^ = 0.00	–	1.00	–
Hereditary	19 (9.9)	30 (15.6)	*χ* ^*2*^ = 2.83	–	.092	–
Chance	41 (21.4)	69 (35.9)	*χ* ^*2*^ = 9.99	–	*.002*	−.16
Stress	160 (83.3)	128 (66.7)	*χ* ^*2*^ = 14.22	–	*.000*	.19
My own behavior	105 (54.7)	65 (33.9)	*χ* ^*2*^ = 16.89	–	*.000*	.21
Others behavior	85 (44.3)	58 (30.2)	*χ* ^*2*^ = 8.12	–	.004	–
Poor medical care	8 (4.2)	38 (19.8)	*χ* ^*2*^ = 22.23	–	*.000*	−.24
Limiting behavior^c^	18.88 ± 4.58	22.43 ± 5.07	*t* = −7.19	−4.52, −2.58	*.000*	−.34
All or nothing behavior	16.59 ± 5.61	14.89 ± 4.68	*t* = 3.17	0.65, 2.76	*.002*	.16
Social support	6.68 ± 1.65	6.60 ± 1.83	*U* = 18388.5	–	.968	–
Depression	6.83 ± 5.29	6.88 ± 5.61	*U* = 18185.5	–	.820	–
Anxiety	6.00 ± 5.22	6.09 ± 5.26	*U* = 18241	–	.860	–
Outcome variables						
Physical functioning	58.09 ± 19.00	35.75 ± 20.26	*t* = 11.15	18.40, 26.28	*.000*	.49
Employment status (*N*, %)			*χ* ^*2*^ = 72.12	–	*.000*	.27
Working, fulltime	63 (32.8)	16 (8.3)				
Working, part-time	81 (42.2)	71 (37.0)				
Not working	27 (14.1)	96 (50.0)				
Studying	21 (10.9)	9 (4.7)				
Medical visits, no.	3.45 ± 3.84	4.49 ± 4.60	*U* = 15145.5	*-*	*.002*	−.16

Values are the mean ± SD unless otherwise indicated. Bonferroni corrected 0.05 alpha level 0.002

*PA* physical activity

^**a**^Confidence interval of the difference

^**b**^More than 20 % of cells had expected count less than 5, an exact significance test was selected for Pearson’s *χ* ^*2*^

^**c**^Levene’s test of equal variance not assumed

In terms of physical, cognitive, behavioral, social, and emotional determinants, univariate differences were found for average hours of sleep per night, minutes of physical activity per week, perceived consequences, timeline, treatment control and identity (illness beliefs), “limiting” and “all or nothing behavior.”. Non-CFS patients more frequently attributed their fatigue to “stress” or “their own behavior” (internal causes) than CFS patients, while CFS patients more frequently attributed their fatigue to “a germ or virus,” “chance,” and “poor medical care in the past” (external and physical causes). No significant differences were found for social support, anxiety, and depression.

With respect to relevant outcomes, the fatigued non-CFS group reported a significantly better physical functioning, a higher employment rate, and less medical visits.

Next, a multivariate logistic regression analysis was conducted to identify which determinants contribute most to the likelihood of being diagnosed with CFS. Only determinants that were significant at a univariate level (see Table 1) were entered as independent variables into the equation. Fatigue duration, fatigue severity, and somatic complaints were not entered as control or independent variables in the equation because they were part of the inclusion criteria used to construct the dependent variable. The “cause” variable of the Brief IPQ was not entered into the regression analysis as patients could indicate multiple causes for their fatigue/CFS. Multicollinearity did not prove to be an issue. The linearity of the logit assumption was tested using the Box-Tidwell test. As physical activity (min/day) (PA) violated the linearity of the logit assumption, a categorical variable for PA was used based on the median. All other variables entered into the model were continuous. Table 2 shows the results of the logistic regression analysis.

**Table 2:** Predictors of chronic fatigue syndrome (*N* = 384)

Predictors	*β*	SE	Wald	OR	95 % CI^b^	*p*
Constant	−5.59	1.04	28.99	.00		.000
Sleep	.043	.084	.268	1.044	0.89-1.23	.605
Min of PA per week (high)^a b^	−.744	.270	7.610	.475	0.28-0.81	*.006*
Consequence	.302	.097	9.682	1.353	1.12-1.64	*.002*
Timeline	.428	.071	36.426	1.530	1.34-1.76	*.000*
Treatment Control	.009	.054	.028	1.009	0.91-1.12	.868
Identity	.170	.073	5.337	1.185	1.03-1.37	*.021*
All or nothing behavior	−.062	.025	5.954	.940	0.90-0.99	*.015*
Limiting behavior	.020	.033	.376	1.020	0.96-1.09	.540

^a^Minutes of physical activity per week

^b^Median minutes of PA per week = 100

^b^Confidence interval of the difference

The regression model was statistically signficant, *χ*^*2*^(8, *N* = 384) = 165.98, *p* < .001), indicating that the model was able to distinguish between the two patient groups. The model explained between 35.1 % (Cox & Snell *R*^*2*^) and 46.8 % (Nagelkerke *R*^*2*^) of the variance in the dependent variable and correctly classified 76.6 % of the cases. Patients with higher consequences, timeline, and identity beliefs were more likely to be classified as having CFS. In contrast, persons with higher levels of PA and higher levels of “All or Nothing” behavior were less likely to be classified as having CFS.

## Discussion

### Main Findings

As expected, CFS patients reported higher levels of fatigue severity, a longer duration of fatigue, and more somatic complaints.

CFS and fatigued non-CFS patients also differed in their causal attribution of fatigue. CFS patients more frequently attributed their fatigue to external and physical causes and non-CFS patients to internal causes. This is in line with the results of other studies [12, 13]. Attribution of symptoms to physical causes has been previously associated with decreased physical activity and higher fatigue in CFS patients, suggesting that this attribution tendency is a maintaining factor [13].

In contrast with the study by Darbishire et al. [9] and Evengård et al. [11], we did not find differences in anxiety and depression between both groups. Differences in measurement instruments and study setting could partly explain this finding. The results of both previous studies could also be subject to a type 1 error, as no statistical correction for multiple comparisons was made.

As expected, CFS patients reported worse physical functioning and a lower employment rate than the fatigued non-CFS group. This is in line with other studies [9, 10]. As in the study by Darbishire et al. [9], CFS patients also paid significantly more visits to their GP and medical specialists.

The results of the multiple logistic regression analysis showed that patients who are more physically active are less likely to be classified as CFS. The amount of sleep per night was not a significant determinant. With regard to illness representations, the results showed that patients who believed that their fatigue was associated with more severe consequences, that it would last for a longer period of time, and patients who attributed more additional symptoms to their condition were more likely to be classified as CFS, a finding that is similar to other studies [9, 16]. Finally, patients who reported lower levels of “all or nothing behavior” were more likely to have a CFS diagnosis. This finding may suggest that “all-or-nothing” behavior is more characteristic of the earlier stages of fatigue, which is in line with a prospective cohort study [16], demonstrating that, within a patient cohort suffering from an acute viral infection, “all-or-nothing” behavior was a strong predictor of the development of CFS over time.

### Limitations and Strengths

A limitation of this study is that the diagnosis of CFS was based on a self-report measure, which could have led to misclassification of some patients. Likewise, the presence of other somatic diseases and/or psychiatric disorders was not ruled out by means of clinical evaluations. The fact that both groups were for the most part recruited in a different way (patient organization for CFS versus primary health care centers for the non-CFS patients) may also have influenced the results. The present study included only quantity of sleep at night. Future studies should also take into account the amount of time spent sleeping during the day, as well as sleep quality, thus allowing for a more complete measurement of the sleep variable. In addition, other potentially important physical determinants such as BMI should also be taken into account in future studies.

Another limitation is the cross-sectional design of the study which does not allow for conclusions about the direction of significant associations. In the present study, there is, e.g., a significant difference in fatigue duration and severity between both study groups, which is, however, most likely due to the inclusion criteria used to distinguish between the CFS and the non-CFS group. In addition, other significant differences that were found (e.g., in physical activity and all-or-nothing behavior) are not necessarily precursors, but could also be consequences of being faced with chronic fatigue. For this reason, future prospective studies are needed to examine whether these factors are indeed risk factors for developing CFS.

An important strength of the present study, compared to other case control studies, is its sample size as well as the fact that the CFS and the fatigued non-CFS patients were matched by age and gender. In addition, this is the first study to include so many potential determinants of a distinction between both groups.

## Conclusion

CFS patients and fatigued non-CFS patients appear to differ in many respects. The differences found in this study with respect to physical, cognitive, and behavioral factors may point at potential risk factors for the transition from medically unexplained fatigue to CFS. A prospective study should explore the predictive value of these risk factors in order to develop a screening instrument that would allow for identifying primary care patients at risk at an early stage in view of the development of interventions.
